# Thickness of melanocytes in giant congenital melanocytic nevus for complete surgical excision: clinicopathological evaluation of 117 lesions according to the area and size

**DOI:** 10.1186/s12893-024-02362-x

**Published:** 2024-03-15

**Authors:** Ji-Young Kim, Se Yeon Lee, Yoonjin Kwak, Byung Jun Kim

**Affiliations:** 1 Department of Plastic and Reconstructive Surgery, Seoul National University College of Medicine, 101 Daehak-ro, Jongno-gu, Seoul, 03080 Republic of Korea; 2 Department of Pathology, Seoul National University College of Medicine, Seoul, Republic of Korea

**Keywords:** Pigmented nevus, Melanocytic nevus syndrome, Malignant transformation, Melanoma

## Abstract

**Background:**

Giant congenital melanocytic nevi (GCMN) are usually defined as nevi that exceed 20 cm in maximal diameter or 15% of the total body surface area. There have been reports of life-long malignant change risks arising from GCMN, leading to surgical excision of GCMN. This study aims to evaluate the thickness of melanocytes based on clinical factors in order to provide objective information for the complete resection of the lesion.

**Methods:**

Overall, 75 patients diagnosed with GCMN between 2000 and 2021 were included, and their clinical records were collected retrospectively. 117 pathologic slides obtained during excision were reviewed to measure nevus thickness. Clinical factors were assessed with a generalized estimated equation model for association with nevus thickness.

**Results:**

The thickness of nevus was significantly associated with the location and size. Nevus thickness was more superficial in the distal extremity than in the head and trunk (*P* = 0.003 [head]; *P* < 0.001 [trunk]; *P* = 0.091 [Proximal extremity]). Nevi sized 60 cm or more were significantly deeper than those measuring 20–29.9 cm (*P* = 0.035). An interaction between size and location existed (*P* < 0.001). Trunk and distal extremity lesions consistently exhibited uniform thickness regardless of lesion size, whereas head and proximal extremity lesions showed variations in thickness based on lesion size.

**Conclusion:**

GCMNs have differences in thickness according to location and size. Therefore, it is necessary to devise an approach optimized for each patient to treat GCMN.

**Mini-abstract:**

In the study, it was emphasized that the thickness of GCMN is correlated with clinical factors, specifically the location and size of the nevus. Consequently, these findings underscore the need for individualized treatment plans for effective surgical intervention.

**Supplementary Information:**

The online version contains supplementary material available at 10.1186/s12893-024-02362-x.

## Introduction

Giant congenital melanocytic nevus (GCMN) usually refers to a nevus with a size of 20 cm or more and has a prevalence of about 1 in 20,000 people [1]. Although GCMN is a sub‑group of congenital melanocytic nevus (CMN), these nevi are deeply located, have a different mutation spectrum, and have an increased risk of malignant transformation. With cosmetic problems, nearly 5% of them reportedly turn malignant [2–4]; therefore, these must be removed. Furthermore, removing the nevus cell deeply enough from an oncologic point of view is important.

Epidemiologic studies on GCMN are readily available, and location, size, satellite lesion, and young age are known as risk factors for the malignant transformation of GCMN [2, 5, 6]. Malignant transformation in GCMN most commonly manifests as melanoma. In melanoma patients, the Breslow thickness, which gauges the depth of the tumor from the skin surface to its deepest point, stands widely recognized as a prognostic factor for survival. This is substantiated by multivariate analyses that reveal heightened hazard ratios corresponding to increasing Breslow thickness [7]. However, there is no detailed information on GCMN thickness or thickness-related factors. In the present study, the actual thickness of the GCMN was measured, and relevant clinical factors [sex, age, height, weight, body mass index (BMI), body surface area (BSA), as well as nevus location and size] were compiled. Furthermore, we aimed to explore possible relationships between these factors and the thickness of GCMN.

## Materials and methods

This retrospective study enrolled patients with GCMN who underwent excision at the Seoul National University Hospital between 2000 and 2021. Patients who underwent physical trauma to the lesion, such as laser or dermabrasion, outside of surgical treatments, were excluded. This decision was made due to the potential for inducing histological differences in the extent and thickness of the nevus. Pathologic slides of the stellate lesions were also excluded. A total of 75 patients with GCMN were included in this study, and 117 slides were reviewed. Ethics approval was obtained from the Seoul National University Hospital Institutional Review Board (Approval No.: 2205-083-1324).

With reference to Krengel et al.’s report [8], patients were divided into four groups according to nevus size (group 1, 20–29.9 cm; group 2, 30–39.9 cm; group 3, 40–59.9 cm; group 4, 60 cm~). The location of the nevus was divided into the head, trunk, proximal extremities, and distal extremities. The distinction between proximal extremities and distal extremities is based on the knee for the lower extremities and the elbow for the upper extremities. For nevi that spanned multiple areas, the primary location was designated based on the location where the slide was obtained. The collected data included demographic information, medical photographs, operation records, nevi characteristics, clinical course, pathology reports, and follow-up period.

### Measurement of nevus thickness

**Fig. 1:**
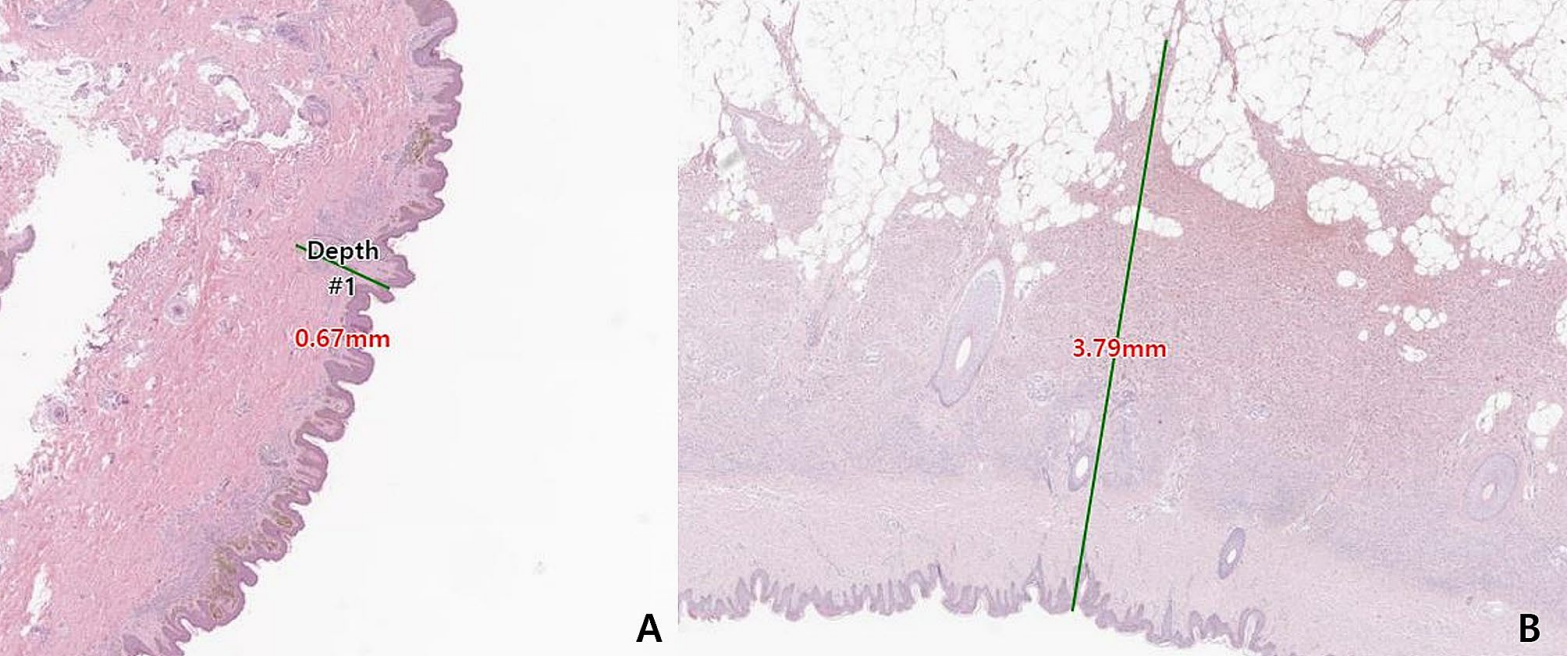
Example of histologic measurement of nevus depth. H&E stained (×40), used for histopathological measurement of infiltration depth in this work. The depth of deepest-seated nevus cells perpendicular to the skin surface is measured. **A**, A 95-month-old female patient underwent surgical excision for a 20–29.9 cm large nevus in the proximal extremity. The thickness of the nevus at a randomly selected point was 0.67 mm. **B**, A 47-month-old male patient underwent surgical excision for a giant nevus sized 30–39.9 cm in the trunk. The thickness of the nevus at randomly selected points is 3.79 mm

Histopathological features were evaluated based on standard H&E stained sections. Four visual fields of each sample were randomly selected for measurement. There is no specialized measurement technique universally applicable to GCMNs. In the case of pigmented lesions such as melanoma, Breslow thickness is commonly used to measure the extent of penetration below the skin surface. Drawing inspiration from this, we established measurement criteria to determine how deeply the nevi penetrate beneath the skin surface. The vertical distance was measured from the top of the epidermis granular layer, including the stratum corneum, to the deepest-seated nevi cell [9]. The thickness of nevi can only be evaluated in sections cut perpendicular to the epidermal surface (Fig. 1). Two independent evaluators collected all measurements in duplicate (1-month intervals between data collection times), and mean values of these observations were used for each analysis.

### Statistical analysis

A total of 117 data sets were generated for the analysis, which was collected using different slides of 75 patients. One to as many as four slides were obtained per patient. The generalized estimating equation (GEE) model was used to verify the significant variables associated with the thickness of the nevus. Further analysis was performed for clinical factors with a p-value of 0.1 or less, considering the interaction between factors. Intra-rater reliability and inter-rater reliability are statistical methods used to assess the reliability of measurement tools. Intra-rater reliability tests reflects the consistency or stability of results when the same measurer performs the same measurement multiple times. Inter-rater reliability tests indicates the consistency or stability of results when different evaluators perform the same measurement. We aimed to assess the consistency of the method for measuring nevus thickness, and intra-rater and inter-rater reliability were analyzed using intraclass correlation coefficients (ICC). For all tests, *p* < 0.05 was considered statistically significant. All statistical analyses were performed using IBM SPSS version 26.0 (IBM Corp., Armonk, NY, Unites States).

## Results

### Clinical features

A total of 75 patients diagnosed with GCMN, comprising 37 males (49.33%) and 38 females (50.67%), were included in this study. A total of 117 pathological specimens were obtained from the study subjects for analysis. Demographic information analysis based on the specimens revealed the inclusion of 58 males (49.57%) and 59 females (50.43%), with ages ranging from 12 months to 25.7 years (mean ± SD age, 64.93 ± 43.88 months). Among the specimens included in the study, those collected from individuals aged 5 years or younger accounted for 66 cases (56.41%), comprising more than half of the total. There were 39 cases (33.33%) in the 6–10 age group, 11 cases (9.40%) in the 11–15 age group. There was one patient aged 16 or older, and the age was 25.7 years. The majority of specimens were obtained from children before the onset of adolescence. The mean height was 109.76 cm (71–164.3 cm). The mean body weight was 21.96 kg (9.1–69.4 kg). The mean BMI was 17.2 kg/m^2^ (13.21–28.05 kg/m^2^). The mean BSA was 0.81m^2^ (0.44–1.74 m^2^). A total of 117 slides were analyzed. The mean nevi thickness of all slides was 3.08 ± 1.22 mm (mean ± SD). The most common location of the nevi was the trunk (62.4%), followed by the proximal extremity (16.2%), distal extremity (12%), and the head (9.4%). Most lesions were 40–59.9 cm in size (32.5%), followed by 30–39.9 cm (27.4%), 20–29.9 cm (23.9%), and over 60 cm (16.2%). The seasons during which specimens were obtained were as follows: 32 cases (27.35%) in spring, 35 cases (29.91%) in summer, 21 cases (17.95%) in autumn, and 29 cases (24.79%) in winter. The intra-rater and inter-rater reliability test results were excellent (ICC = 0.893–0.922) for thickness measurements.

### Factors influencing nevus thickness

**Fig. 2:**
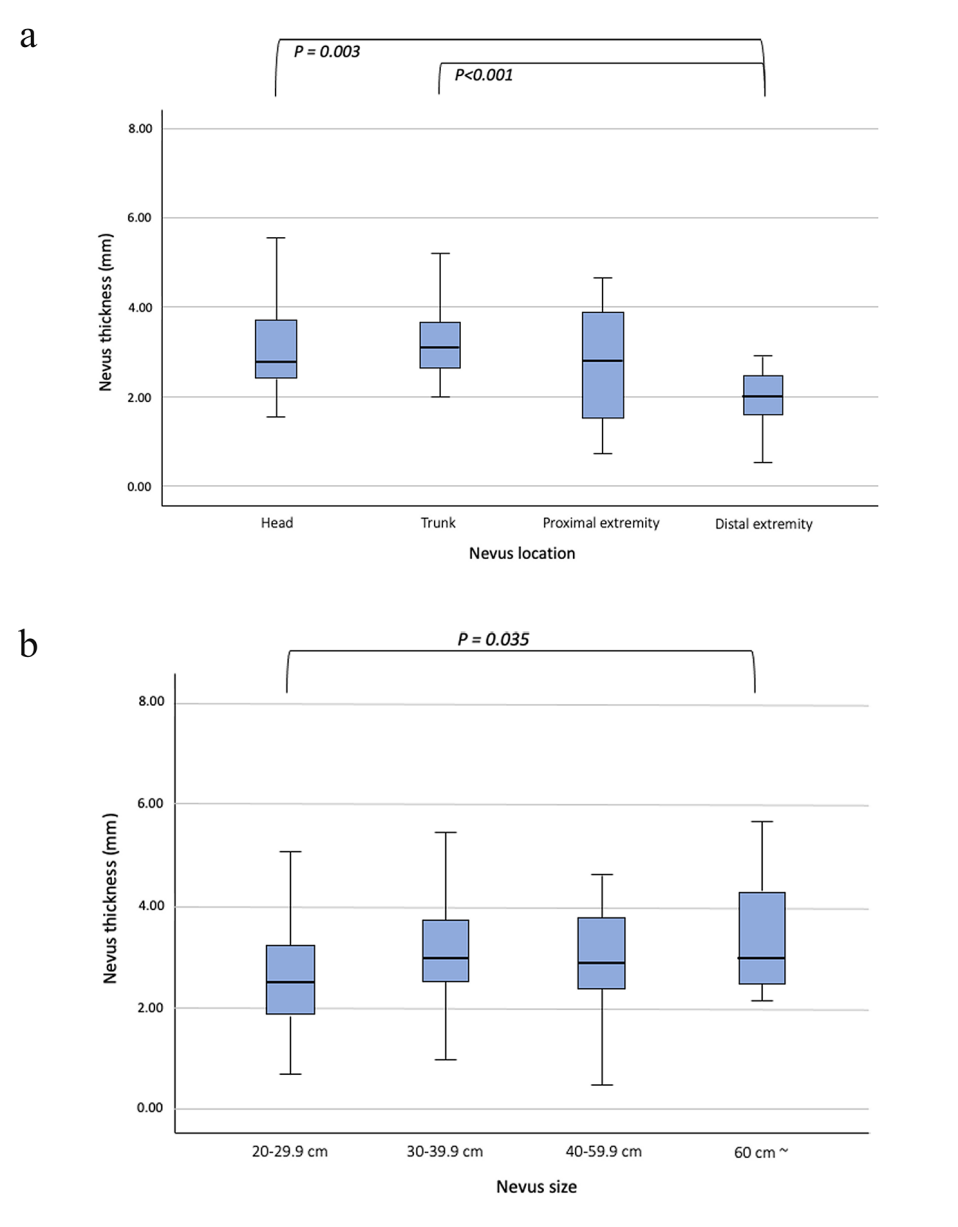
Difference of nevus thickness according to location and size. **A**, Nevus thickness according to nevus location. **B**, Nevus thickness according to nevus size

In a GEE analysis, size and location were significantly associated with the thickness of nevus. Differences in nevus thickness according to sex, age, height, weight, BMI, BSA, season, size, and location are shown in Table S1 (Supplemental Digital Content). Nevus thickness tended to be more superficial in the distal extremity (1.97 ± 0.70 mm) than in the head, trunk, and proximal extremity (3.23 ± 1.25 mm; *P* = 0.003 [head]; 3.37 ± 1.13 mm; *P* < 0.001 [trunk]; 2.69 ± 1.33 mm; *p* = 0.091 [Proximal extremity], respectively) (Fig. 2A). Nevus thickness was also significantly deeper for the sizes of over 60 cm (3.54 ± 1.37 mm) than in the size of 20–29.9 cm (2.65 ± 1.15 mm; *P* = 0.035) (Fig. 2B). Other factors (sex, age, height, weight, BMI, BSA and season) were not significantly associated with nevus thickness.

### Interaction between Nevus size and location for nevus thickness

**Fig. 3:**
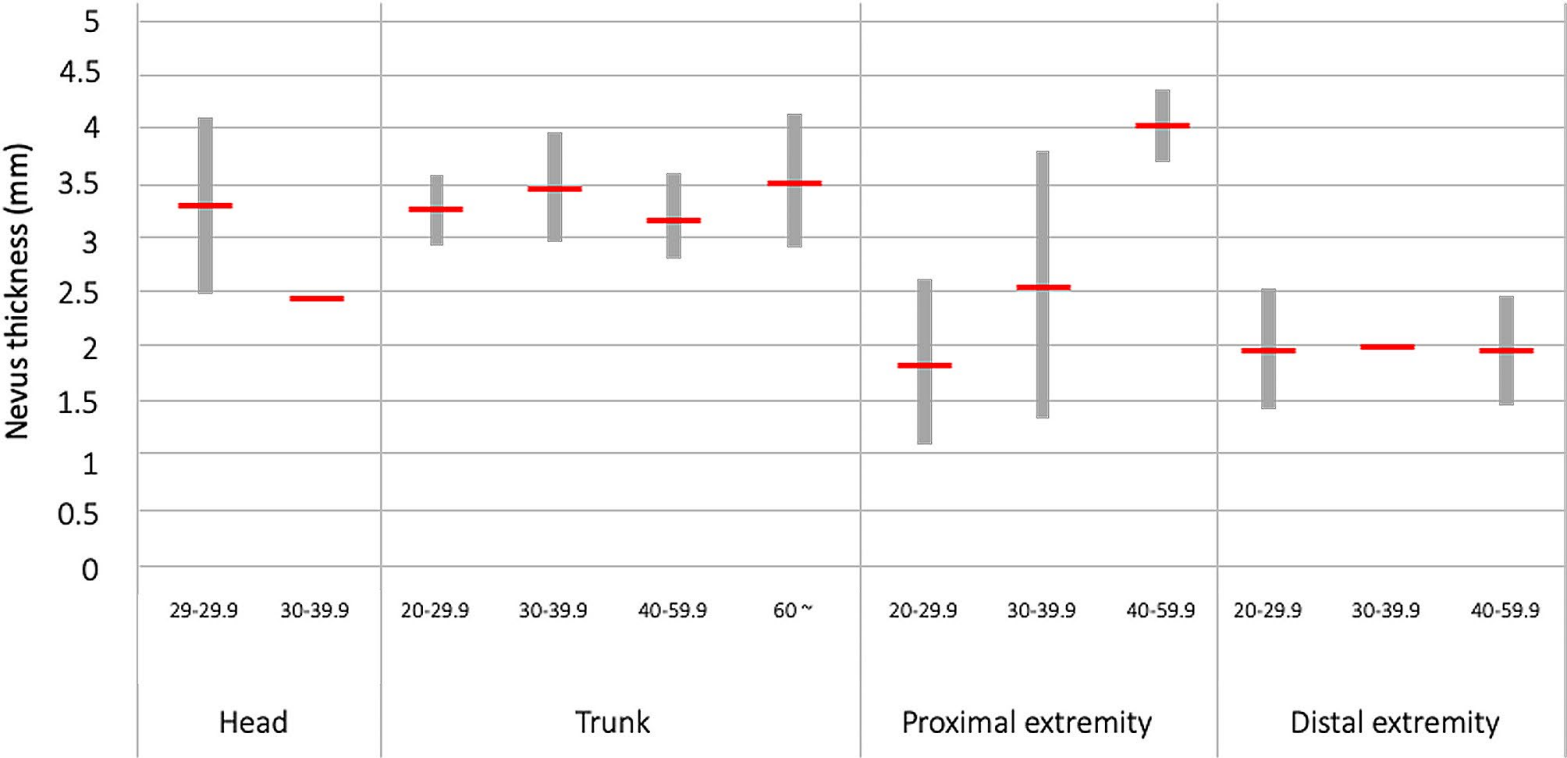
Estimated average thickness of GCMN in consideration with the size and location

The factors that showed a significant relationship with the nevus thickness were the size and location of nevus. Based on this, an analysis was conducted to examine how the size and location of nevus, as well as the interaction between size and location, independently influence the thickness. There was a significant interaction between size and location (*p* < 0.001). The results of the average estimate obtained considering both the size and location of the nevus are summarized in Fig. 3. The nevus with a size of 60 cm or more were exclusively located on the trunk, and there were no lesion with a size of 40 cm or larger on the head. Post-hoc analysis was conducted to examine whether differences in nevus size based on the location of nevus have an impact on nevus thickness. There were no significant differences in nevus thickness based on size for distal extremity and trunk lesion. Regardless of the size, nevi located on the trunk exhibited thicker lesions, while nevi on the distal extremity appeared superficial. In the case of proximal extremity, there was a tendency for thicker lesion with larger sizes, and the nevus measuring 40–59.9 cm were significantly thicker than those measuring 20–29.9 cm (*p* < 0.001). For head lesions, a significant difference in thickness was observed between the size of 20–29.9 cm and 30–39.9 cm (*P* = 0.041).

## Discussion

The current information about GCMN is lacking, and previous studies have mainly focused on the epidemiologic characteristics of GCMN [10–13]. In malignant melanoma, which is the most common type of malignant transformation of GCMN, Breslow thickness has been known to be the most important prognostic factor for survival [14]. Previous studies have shown that the thickness of cutaneous melanoma is associated with a BMI of ≥ 25 kg m^2^ [15, 16]. Higher Breslow thickness was also associated to scalp localization of cuataneous Melanoma [17]. These suggest that the thickness of the melanocytic lesion on the skin may be related to anthropometric factors. To the best of our knowledge, this is the first study to examine GCMN thickness as a quantitative outcome according to clinical factors. In the present study, clinical factors related to the thickness of the giant nevus were the location and size of the nevus. However, there was only one case of malignant transformation among the patients included in this study, and it was not possible to determine the relationship between the thickness of the nevus and the prognosis, like the Breslow thickness.

Most GCMNs, unlike CMN, histologically have a sub-epidermal non-involvement zone below the epidermis. As for the infiltration of nevus cells, it was observed that they infiltrated deeply throughout the dermis and even into the subcutaneous adipose tissue. These histological features of GCMN are related to the mechanism of nevus development during the embryonic period. At the embryonic stage, melanoblasts migrate from the neural crest, leading to nevus formation. Depending on the time of mutation, the clinical and pathological characteristics are also affected. When mutations occur earlier, they appear larger and more deeply invaded. Since there is a specific distance between the epidermis and proliferated nevus cells, a nevus cell-poor zone is formed below the epidermis [18]. In this study, nevi sized 60 cm or more were significantly deeper than those measuring 20–29.9 cm, consistent with prior findings.

Skin thickness varies according to the anatomical site [19, 20]. According to one study, the thickness of the epidermis was most affected by the body site [21]. Lee Y et al. [20] suggested that the skin of the back was the thickest, and in the case of the palm and soles, the epidermis was the thickest, and the proportion of the epidermis in the entire skin was higher than that of other parts. Since mutation occurs during the process of melanoblasts originating from the neural crest ascending to the skin, we hypothesized that variations in skin thickness across anatomical sites would influence nevus thickness. In this study, we found that the average thickness of the nevus was the thickest in the trunk, including the back, and the nevus thickness of the distal extremity was significantly more superficial than that of the head and trunk.

However, it is important to consider that the two factors of nevus size and nevus location cannot be considered independently. The distribution of nevus has been discussed previously in several articles. Sahin et al. [22] examined only medium-sized CMNs and found that the head and neck were the most common site of nevus, followed by the trunk. Yun et al. [23] and Kim et al. [24] reported that GCMN was located primarily in the trunk. The proportion of each part of the body in the total BSA was consistent [25], and probabilistically large nevus tended to belong to a large area of our body (i.e., trunk). In the present cohort of GCMN cases, the most common location was on the trunk (62.4%), followed by the proximal extremity (16.2%), distal extremities (12%), and head (9.4%). The case of the head with the largest nevi belonged to the 30–39.9 cm group, and all giant nevus over 60 cm were in the trunk. This suggests that the distribution of nevus locations may vary depending on the size of the nevus. In addition, the results showed that the interaction between location and size affects the thickness of the nevus. Unlike head and proximal extremity lesions, trunk and distal extremity lesions were less influenced by nevus size in terms of nevus thickness. Even if they are classified as the same location, there may be differences in skin thickness depending on the side (anterior/posterior or dorsal/palmar). In the case of the head, within a relatively small area, there are many variations in the skin’s characteristics, depending on whether the nevus is dominant on the scalp or face. This could potentially influence the thickness. For distal extremities, the influence may be less significant since slides of palms and soles were not included. Categorization of the location in this study does not account for the characteristics of all areas of the skin due to the lack of a sample number. A study is needed in the future to obtain and analyze additional samples. Nevertheless, the tendency of the overall thickness according to the location could be seen.

**Fig. 4:**
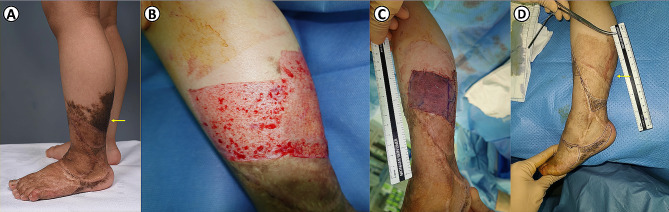
The GCMN of the lower leg was excised with a dermatome blade. **A**, Preoperative photograph. **B**, After excision with a dermatome. **C**, Immediate postoperative photograph. After excision, split-thickness skin grafting was performed. **D**, Postoperative 7-month photograph

Surgical treatment is a definitive treatment to eliminate the nevi. However, depending on the location (i.e., hand, foot, and face), it is difficult to eradicate the nevi completely, and the operation must be repeated several times because of the extensiveness of the lesion. Therefore, non-surgical methods such as chemical peeling and laser therapy are also used to treat GCMN. Although laser therapy is reportedly effective in removing small melanocytic nevi [26], it only targets the superficial portion of the nevus that carries pigments, and subcutaneous nevus cells are left behind and covered with a layer of superficial scar tissue [18]. The remnant nevus cells in the deep layer still possess a malignant potential; melanoma transformation in GCMN has been reported after non-surgical treatment [27, 28] or even surgical excision [29]. Therefore, caution is warranted when selecting a treatment method for GCMN. Knowing more specific quantitative information about the GCMN thickness will help select and apply an appropriate method based on location and size. It can also be used as data to develop a new removal strategy. For example, the thickness of the nevus in the distal extremity is the most superficial, and rapid and standardized resection using a dermatome could be attempted (Fig. 4).

Our study had some limitations. Because the sample size was not large enough, the classification of the location does not reflect the pathological characteristics of the skin from all anatomical sites, and only the overall trend could be observed. In the case of the bottom surfaces of the hands and feet, for cosmetic and functional reasons, surgical excision is performed at a relatively later stage or just observed without removal. This study did not include slides on these cases; therefore, information about them could not be obtained. Lastly, with only one slide of a patient with malignant transformation (melanoma), it remains unknown whether the thickness affects the prognosis of GCMN patients. Larger scale studies with a more detailed classification of location are necessary to broaden the knowledge on this topic.

## Conclusion

In GCMN, the location and the size of the nevus affect its thickness. Efforts should be made to provide personalized treatment by considering these facts in the treatment approach for children with GCMN.

### Electronic supplementary material

Below is the link to the electronic supplementary material.

Supplementary Material 1: Table S1. Difference in the nevus thickness according to sex, age, height, weight, BMI, BSA, season, location, and size, (BMI, body mass index; BSA, body surface area).

## Data Availability

All data generated or analyzed during this study are included in this published article. The datasets used in the current study are available from the first author upon reasonable request.
