# Supplementary material for: Thickness of melanocytes in giant congenital melanocytic nevus for complete surgical excision: clinicopathological evaluation of 117 lesions according to the area and size
Source: BMC Surg. 2024 Mar 15;24:90. doi: 10.1186/s12893-024-02362-x (PMC10941407; doi:10.1186/s12893-024-02362-x)
Supplement: Supplementary file 1 — Supplementary Material 1: Table S1. Difference in the nevus thickness according to sex, age, height, weight, BMI, BSA, season, location, and size, (BMI, body mass index; BSA, body surface area). [file 12893_2024_2362_MOESM1_ESM.docx]

Table S1. Difference in the nevus thickness according to sex, age, height, weight, BMI, BSA, season, size, and location (BMI, body mass index; BSA, body surface area)

| **Variables** | **No. of slides (n, %)** | **Nevus thickness (Mean±SD, ㎛)** | **Regression coefficient B** | **Standard error** | **p-value** |
| --- | --- | --- | --- | --- | --- |
| **Sex** |  |  |  |  |  |
| Male | 58 (49.57) | 2.96±1.24 | -0.242 | 0.509 | 0.351 |
| Female | 59 (50.43) | 3.20±1.19 |  |  |  |
| **Age, years** | 117 | 64.93±43.88 | 0.003 | 0.006 | 0.314 |
| **Height, cm** | 117 | 109.76±21.17 | 0.007 | 0.012 | 0.24 |
| **Weight, kg** | 117 | 21.96±11.28 | 0.012 | 0.024 | 0.321 |
| **BMI, kg/m^2^** | 117 | 17.20±2.17 | 0.055 | 0.148 | 0.464 |
| **BSA, m^2^** | 117 | 0.81±0.27 | 0.544 | 0.948 | 0.261 |
| **Season** |  |  |  |  |  |
| Spring | 32 (27.35) | 3.03±1.08 | -0.424 | 0.503 | 0.098 |
| Summer | 35 (29.91) | 3.00±1.30 | -0.46 | 0.599 | 0.133 |
| Autumn | 21 (17.95) | 2.76±1.33 | -0.699 | 0.744 | 0.066 |
| Winter | 29 (24.79) | 3.46±1.22 | Reference |  |  |
| **Nevus size** |  |  |  |  |  |
| 20-29.9 cm | 28 (23.9) | 2.65±1.15 | -0.891 | 0.826 | 0.035 |
| 30-39.9 cm | 32 (27.4) | 3.18±1.30 | -0.361 | 0.794 | 0.373 |
| 40-59.9 cm | 38 (32.5) | 3.08±1.04 | -0.465 | 0.752 | 0.225 |
| 60~ cm | 19 (16.2) | 3.54±1.37 | Reference |  |  |
| **Location** |  |  |  |  |  |
| Trunk | 73 (62.4) | 3.37±1.13 | 1.402 | 0.434 | <0.001 |
| Head | 11 (9.4) | 3.23±1.25 | 1.262 | 0.846 | 0.003 |
| Proximal extremity | 19 (16.2) | 2.69±1.33 | 0.72 | 0.835 | 0.091 |
| Distal extremity | 14 (12) | 1.97±0.70 | Reference |  |  |
